# Relationship between the lesion location of acute ischemic stroke and early depressive symptoms in Japanese patients

**DOI:** 10.1186/s12991-016-0099-x

**Published:** 2016-04-01

**Authors:** Norifumi Metoki, Norio Sugawara, Joji Hagii, Shin Saito, Hiroshi Shiroto, Tetsu Tomita, Minoru Yasujima, Ken Okumura, Norio Yasui-Furukori

**Affiliations:** Hirosaki Stroke and Rehabilitation Center, Hirosaki, Japan; Department of Neuropsychiatry, Hirosaki University School of Medicine, 5 Zaifu-cho, Hirosaki, 036-8562 Aomori Japan; Department of Cardiology, Hirosaki University School of Medicine, Hirosaki, Japan

## Abstract

**Background:**

Approximately one-third of stroke survivors suffer from post-stroke depression (PSD) in the acute or chronic stages. The presence of PSD in the acute stage after stroke is reportedly associated with poor patient prognosis; therefore, early recognition and treatment of PSD may alleviate these consequences. The aim of the current study was to examine the relationship between the lesion location and the presence of early depressive symptoms after acute ischemic stroke in Japanese patients.

**Methods:**

Our study included 421 patients who suffered from acute ischemic stroke. On the day of admission, the lesion location was determined using magnetic resonance imaging (MRI). Stroke severity was measured on the seventh day of hospitalization withat the National Institutes of Health Stroke Scale (NIHSS). On the tenth day of hospitalization, depressive symptoms were measured and functional assessments were performed with the Japan Stroke Scale (Depression Scale) (JSS-D) and functional independence measure (FIM), respectively.

**Results:**

A total of 71 subjects (16.9 %) were diagnosed with depression. According to the multiple logistic regression analysis, the infarcts located at frontal and temporal lobes were found to be a significant independent risk factor of early depressive symptoms in the acute stage of stroke.

**Conclusions:**

Patients suffering from acute ischemic stroke, particularly in the frontal and temporal lobes, should be carefully assessed to detect and treat early depressive symptoms; such treatment may improve patient outcomes.

## Background

Post-stroke depression (PSD) is a serious neuropsychiatric complication with a high rate of prevalence after stroke. Approximately one-third of stroke survivors suffer from early or late depressive symptoms after the onset of stroke [1]. Because PSD symptoms are related to increased functional disability [2] and poorer rehabilitation outcomes [3], the factors associated with PSD are of great clinical importance.

Compared with patients who develop depressive symptoms later, patients who develop PSD during the acute stage of stroke demonstrate more somatic signs of depression rather than endogenous signs of depression [4], and they more frequently show early signs of melancholy, vegetative state, and psychological disturbance with poor social skills [5]. In addition, several longitudinal studies have shown that the incidence of PSD is higher in the first month post-stroke [6–8] compared with later in the first year after stroke. Although it is difficult to prevent stroke, the early screening and treatment of PSD in the acute stage may be the key to alleviating its long-term consequences.

In addition to psychological reactions due to disability, loss of independence, and impaired quality of life (QOL) [9], pathophysiological factors, such as changes in neurotransmitters [10] and the specific site of infarcts [11], have also been hypothesized to be related to the development of PSD. Indeed, the relationship between the stroke lesion locations and PSD has been the topic of much research [12]. Although many studies of PSD, including meta-analyses, have been reported, the association between the lesion location and the occurrence of PSD remains unclear [13–15], likely because of the variables, such as time post-stroke, and the type of instruments used to assess PSD.

The objectives of this investigation were (1) to evaluate the prevalence of early depressive symptoms among patients with acute ischemic stroke and (2) to examine the relationship between the lesion location and presence of early depressive symptoms. To our knowledge, this study is the largest to date in a Japanese population.

## Methods

### Participants

We reviewed the charts of 532 patients who were admitted for acute stroke between April 2012 and March 2013 at Hirosaki Stroke and Rehabilitation Center. The stroke diagnosis was based on both the presence of acute neurological symptoms and a compatible lesion on magnetic resonance imaging (MRI) on the first day of hospitalization. We excluded patients with (1) serious comprehension difficulties (e.g., severe aphasia), (2) hemorrhage, and (3) a history of psychiatric disease (e.g., depression, dementia, etc.). Psychiatric histories were obtained from medical records based on interviewing patients and family. After a detailed evaluation with inclusion and exclusion criteria, 421 patients who suffered from acute ischemic stroke participated in this study. The following information was collected for each patient: demographics (i.e., age, gender, education level, and living alone) and stroke severity, as measured with the National Institutes of Health Stroke Scale (NIHSS) on the seventh day of hospitalization [16]. Assessment of NIHSS scale was performed by trained stroke nurses, and the testing was confirmed by neurologists.

The data collection for this study was approved by the Ethics Committee of the Hirosaki University School of Medicine and Hirosaki Stroke and Rehabilitation Center. Informed consent was obtained from all patients before the study.

### Assessment of early depressive symptoms and functional independence

The Japan Stroke Scale (Depression Scale) (JSS-D), which was developed by the Japan Stroke Society, was administered to all participants to measure their depressive status [17]. The JSS-D is a 7-item (i.e., mood, guilt feelings, interest, apathy, anxiety, sleeplessness, and expression) objective measurement, in which three choices are given for each item. The total score, which indicates the degree of depression, was calculated based on the proper weight of each choice. Based on ROC curve analysis of JSS-D, probable PSD was defined as a score of 2.4 or higher. The sensitivity and specificity of this cut-off score were 0.950 and 0.988, respectively [17]. In this study, JSS-D data were collected on the tenth day of hospitalization to assess depressive symptoms in acute post-stroke phase (≤1 month). Assessment of JSS-D was performed by trained stroke nurses, and the testing was confirmed by psychiatrists.

Functional independence was assessed with the 18-item functional independence measure (FIM), which consists of six domains: self-care, sphincter control, mobility, locomotion, communication, and social cognition. The FIM scores a patient’s dependence level from 18 (total assistance in all areas) to 126 (complete independence in all areas) [18] and were administered on the tenth day of hospitalization. Assessment of FIM was performed by trained stroke nurses, and the testing was confirmed by neurologists.

### Brain imaging

We performed MRI (Signa EXCITE HD 1.5T; GE Medical Systems, Waukesha, USA), including transversal diffusion weighted imaging (DWI), T2-weighted imaging, and fluid-attenuated inversion recovery (FLAIR). In terms of the lesion classifications, information about the infarct site included the affected brain lobes (i.e., frontal, temporal, parietal, and occipital lobe) and the specific anatomical structure/location (i.e., thalamus, caudate, putamen, anterior and posterior limb of internal capsule, corona radiata, centrum semiovale, hippocampus, and amygdala). Stroke specialist physicians certified by Japan stroke society analyzed the images.

### Statistical analysis

Descriptive analyses of the demographic and clinical variables were performed. To perform between-group comparisons of the main demographic and clinical characteristics of the patients, an unpaired Student’s *t* test was performed to analyze continuous variables, and a Chi-squared test or Fisher’s exact test was performed to analyze categorical variables. Data are presented as the mean ± SD. All variables with *p* < 0.10 in the univariate analysis were subsequently analyzed by multivariate logistic regression. For confounding factors, including age, gender, education level, living alone, and NIHSS and FIM scores, multivariate logistic regression analysis was applied to assess the relationship between the lesion location and probable PSD, with a significance level of *p* < 0.05. Data were analyzed using the PASW Statistics PC software for Windows, version 18.0.0 (SPSS Inc., Chicago, IL, USA).

## Results

### Patient characteristics

The mean JSS-D score was 1.53 ± 1.62 for males and 1.77 ± 2.24 for females (*p* > 0.05). The prevalence of probable PSD (based on a JSS-D cut-off of 2.4) (17) was 16.3 % in males (*n* = 43) and 17.8 % in females (*n* = 28) (*p* > 0.05). Table 1 shows the characteristics of the subjects with and without depressive symptoms.

**Table 1:** Characteristics according to subjects with and without depressive symptoms

	Subjects with probable PSD	Subjects without probable PSD	*p* value
*n*	71	350	
Age	74.4 ± 11.1	71.6 ± 11.2	0.052
Gender	Male 43, Female 28	Male 221, Female 129	0.682
Amount of education (year)	9.9 ± 2.8	9.8 ± 2.5	0.936
Living alone	10	54	0.767
NIHSS total score	6.3 ± 5.2	2.7 ± 3.4	<0.001
FIM total score	72.7 ± 34.2	102.2 ± 28.4	<0.001

*PSD* post-stroke depression, *NIHSS* National institutes of health stroke scale, *FIM* functional independence measure

### Logistic regression analysis

The lesion locations in patients with and without depressive symptoms are shown in Table 2. The infarct more frequently affected the following locations in the probable PSD group compared with the non-PSD group: the frontal lobe (*p* < 0.001), temporal lobe (*p* < 0.001), parietal lobe (*p* = 0.011), and putamen (*p* = 0.011), when both sides were considered. To examine the potential independent radiological factors for the development of probable PSD, we included radiological findings with *p* < 0.10 from the univariate analysis in a multiple logistic regression model, which used adjusted factors including age, gender, and post-stroke NIHSS and FIM scores. This analysis suggested that independent radiological risk factors for probable PSD might include infarcts located at the frontal and temporal lobes (Table 3).

**Table 2:** Lesion location in patients with and without depressive symptoms

	Subjects with probable PSD (*n* = 71)		Subjects without probable PSD (*n* = 350)		*p* value
	*n*	%	*n*	%	
Frontal lobe					
Any side	18	25.4	30	8.6	<0.001
Left side	7	9.9	14	4.0	0.065
Right side	11	15.5	16	4.6	<0.01
Temporal lobe					
Any side	18	25.4	28	8.0	<0.001
Left side	7	9.9	12	3.4	0.027
Right side	11	15.5	16	4.6	0.002
Parietal lobe					
Any side	18	25.4	47	13.4	0.011
Left side	9	12.7	21	6.0	0.046
Right side	10	14.1	33	9.4	0.238
Occipital lobe					
Any side	10	14.1	37	10.6	0.391
Left side	3	4.2	16	4.6	1.000
Right side	7	9.9	22	6.3	0.302
Thalamus					
Any side	6	8.5	42	12.0	0.391
Left side	2	2.8	20	5.7	0.556
Right side	4	5.6	24	6.9	1.000
Caudate					
Any side	6	8.5	13	3.7	0.109
Left side	3	4.2	9	2.6	0.434
Right side	3	4.2	4	1.1	0.097
Putamen					
Any side	18	25.4	47	13.4	0.011
Left side	7	9.9	16	4.6	0.086
Right side	11	15.5	31	8.9	0.089
Anterior limb of internal capsule					
Any side	6	8.5	13	3.7	0.109
Left side	3	4.2	7	2.0	0.383
Right side	3	4.2	6	1.7	0.181
Posterior limb of internal capsule					
Any side	6	8.5	33	9.4	0.796
Left side	2	2.8	16	4.6	0.505
Right side	4	5.6	17	4.9	0.766
Corona radiata					
Any side	23	32.4	111	31.7	0.911
Left side	9	12.7	61	17.4	0.327
Right side	14	19.7	50	14.3	0.245
Centrum semiovale					
Any side	12	16.9	47	13.4	0.442
Left side	5	7.0	20	5.7	0.591
Right side	7	9.9	28	8.0	0.605
Hippocampus					
Any side	3	4.2	15	4.3	0.982
Left side	2	2.8	7	2.0	0.652
Right side	2	2.8	8	2.3	0.679
Amygdala					
Any side	0	0.0	5	1.4	0.595
Left side	0	0.0	1	0.3	1.000
Right side	0	0.0	4	1.1	1.000

*PSD* post-stroke depression

**Table 3:** Lesion locations associated with having depressive symptoms estimated by logistic regression analysis

Lesions	Adjusted odds ratio	95 % Confidence interval	*p* value
Frontal lobe			
Any side	2.534	1.188–5.403	0.016
Left side	1.849	0.607–5.636	0.279
Right side	2.709	1.057–6.946	0.038
Temporal lobe			
Any side	3.082	1.454–6.535	0.003
Left side	2.363	0.794–7.038	0.122
Right side	3.093	1.212–7.895	0.018
Parietal lobe			
Any side	1.500	0.739–3.045	0.262
Left side	2.201	0.871–5.565	0.095
Caudate			
Right side	6.936	1.328–36.237	0.022
Putamen			
Any side	1.933	0.956–3.908	0.067
Left side	1.795	0.603–5.339	0.293
Right side	1.803	0.782–4.156	0.166

Adjusted by age, gender, amount of education, living status, NIHSS, and FIM total score *NIHSS* National institutes of health stroke scale, *FIM* functional independence measure

## Discussion

In this study, the prevalence of PSD (based on the JSS-D score) was 16.9 % in the early stroke period. The prevalence of PSD in our study was within the range of previous results, ranging from 6 to 37 % [19–22]. This study of lesion locations demonstrated that frontal and temporal lobe area infarctions are significantly and independently associated with depressive symptoms at the tenth day of hospitalization.

In the 1980s, Robinson and colleagues [23] first showed that depression scores based on both Zung and Hamilton rating scales were significantly higher in patients with left frontal lesions than in patients with lesions in any other location, indicating that there may be an association between specific lesion locations and mood disorder in patients who suffered from acute stroke. Subsequently, several studies [24–27] have also shown that PSD was associated with lesions in the frontal lobes, basal ganglia, and temporal lobes. Recent studies have further demonstrated a relationship between the limbic-cortical-striatal-pallidal-thalamic (LCSPT) circuit and the pathophysiology of major depressive disorder [28, 29]. Terroni and colleagues have reported that among patients with ischemic stroke, larger lesions in the left cortical regions of the LCSPT circuit are associated with a higher incidence of major depressive episode [30]. Together, these findings support our results and indicate that ischemic lesions in certain areas can increase the risk of PSD. However, our results concerning hemispheric lateralization should be cautiously interpreted, because a recent meta-analysis observed that hemispheric laterality of ischemic lesions was not associated with the onset of PSD in an acute post-stroke subgroup [11].

To date, the depletion of biogenic amine neurotransmitters or alterations in their post-synaptic receptor sensitivities have been thought to play important roles in the development of PSD [10], and several studies have investigated medication for PSD [31, 32]. However, it should be noted that many PSD patients may not receive effective treatment for their mood disorders. To improve the long-term outcomes of stroke, it is important to provide early screening and treatment for PSD in the acute stage.

One strength of this study was the use of MRI to assess the infarct site. Many previous studies of PSD have used computed tomography (CT), whereas few studies in this field have used MRI. For detection of acute ischemic stroke, MRI provides greater image resolution, which can identify the site and extent of the infarct with more sensitivity than CT [33, 34].

The current findings must be cautiously interpreted for the following reasons. First, this study used only one measure of depressive symptoms, the JSS-D, and patients were not diagnosed with clinical depression using the Diagnostic and Statistical Manual of Mental Disorders, Fourth Edition. Although JSS-D screening has been well validated and is widely used to assess depressive symptoms, this tool is not a formal diagnostic tool for depression. In addition, the PSD assessment was conducted on the tenth day of hospitalization. Although half of the median reported prehospital delays ranging from 3 to 4 h [35], we cannot completely rule out delays from onset to admission. Second, lesion size was not assessed in this study. Although some studies have not shown an association between lesion size and PSD [24, 36], other studies have reported that lesion size could affect the development of PSD [25, 37]. Third, some important factors, such as medical complications and medication, were not assessed by our study. Finally, we excluded subjects with severe comprehension difficulties because they could not complete the evaluation, which could limit the generalization of our findings.

## Conclusions

Our study found that the prevalence of PSD based on JSS-D was 17 %, which is in agreement with comparable studies. Patients who have suffered from ischemic stroke, particularly in the frontal and temporal lobes, should be carefully evaluated for early detection and treatment of depressive symptoms.
